# Thermal processing and allergenic potential of egg for children

**DOI:** 10.1186/2045-7022-5-S3-P134

**Published:** 2015-03-30

**Authors:** Chantal Brossard, Marie-Anne Legoux, Julie Chabauty, Fabienne Rancé, Agnès Juchet, Martine Drouet, Evelyne Paty, Sandrine Legoué-Morillon, Françoise Nau, Olivier Tranquet, Chantal Brossard

**Affiliations:** 1 Institut National de la Recherche Agronomique, UR1268 BIA, Nantes, France; 2 CHU Toulouse - Pediatrie - Pneumologie, Allergologie, Toulouse, France; 3 CHU d'Angers - Departement Pneumologie - Unite Allergologie Generale, Angers, France; 4 CHU Necker, Pneumo Allergologie Infantile, Paris, France; 5 CHU de Nantes - Centre de Recherche et d'Education en Allergie Alimentaire, Nantes, France; 6 UMR1253 INRA/AgroCampus Ouest, Sciences et Technologie du Lait et de l’ oeuf, Rennes, France

## 

Egg belongs to the list of ingredients that must be labeled because of its frequent involvement in food allergies especially in childhood. Several thermal processes are commonly applied to egg and it is known that some egg-allergic people can tolerate well-cooked egg. Food allergens are characterized by their ability to elicit IgE antibodies (sensitization) in susceptible individuals and to display several IgE-binding epitopes. Allergenic potential influenced by different structural levels (sequence, 2D/3D structures as well as supra-molecular organization) can be greatly impacted by all modifications of these structures due to processing. Our work aimed to analyze the impact of pasteurization and boiling of egg and its fractions on in vivo reactivity and its relation to IgE-binding profiles to egg allergens/fractions in a cohort of children allergic to egg.

Allergic children (n=52, 18 months - 7 years old) were recruited, all had positive skin prick-tests (SPT) to raw whole egg and egg white and 80% to raw egg yolk. Reactivity to pasteurized and boiled fractions was evaluated by SPT. Sera samples taken at inclusion were used to draw profiles of IgE-reactivity against known allergens and proteins of egg white and against fractions of egg yolk by ELISA. Relative STP data and IgE-binding profiles were analyzed by hierarchical classification to evidence typical profiles of reactivity in this cohort.

Pasteurization had very limited impact on structure of egg and only 10% and 5% of children STP became negative with pasteurized (66°C, 6 min) whole egg or egg white. Boiling (100°C, 10 min) greatly changed structure of egg products and had a higher effect than pasteurization since 55%, 27% and 67% of children SPT remained positive to boiled egg and yolk and white fractions. Two typical STP behaviors were evidenced by classification in this cohort. IgE-binding profiles largely differed among children but three typical profiles where extracted from data. Contingency analysis revealed links between typical STP and IgE-binding profiles with possible explanation of tolerance to cooked egg.

